# The promise of circulating tumor cell analysis in cancer management

**DOI:** 10.1186/s13059-014-0448-5

**Published:** 2014-08-30

**Authors:** Joaquin Mateo, Marco Gerlinger, Daniel Nava Rodrigues, Johann S de Bono

**Affiliations:** Division of Cancer Therapeutics and Division of Clinical Studies, The Institute of Cancer Research, 15 Cotswold Road, Sutton, Surrey, SM2 5NG UK; Drug Development Unit, The Royal Marsden NHS Foundation Trust, Downs Road, Sutton, Surrey, SM2 5PT UK; Centre for Evolution and Cancer, The Institute of Cancer Research, 123 Old Brompton Road, London, SW7 3RP UK; Gastrointestinal Cancer Unit, Department of Medicine, The Royal Marsden NHS Foundation Trust, Fulham Road, London, SW3 6JJ UK

## Abstract

Enumeration and molecular characterization of circulating tumor cells isolated from peripheral blood of patients with cancer can aid selection of targeted therapy for patients, monitoring of response to therapies and optimization of drug development, while also providing valuable information about intratumoral heterogeneity.

## Introduction

With the advent of therapies that target the specific molecular aberrations driving a cancer, the traditional concept of ‘one treatment fits all’ is evolving to ‘one patient, one treatment’. Furthermore, as tumors adapt to the selection pressures of serial targeted drugs through the evolution of drug-resistant clones, their genomic landscape changes over time, and hence treatments need to be tailored accordingly [1]. Thus, treatment strategies will almost invariably progress further to ‘one patient at one moment in time, one treatment’.

The endeavor for precision medicine demands that each patient be molecularly characterized and that robust and validated ‘real-time’ assays are made in order to evaluate tumor evolution. Although the study of tumor biopsies or surgical specimens remains the ‘gold standard’ for molecular characterization in clinical trials testing a biomarker [2,3], single-sample analyses fail to represent the heterogeneous genomic evolution of tumors [4]. Considering the physical, logistical and ethical limitations of repeating multiple tumor biopsies in patients, biomarkers that could be judged through minimally invasive procedures, such as blood draws, constitute an opportunity for progression in precision medicine.

Circulating tumor cells (CTCs), which are shed into the bloodstream from solid tumors, are relatively rare, representing only one in more than a million blood cells [5]. Patients with metastatic cancer are more likely to have detectable CTCs in the bloodstream [6], but CTCs also exist in patients with localized disease, even after primary radical treatment, when their presence is informative of recurrence risk [7]. Although the presence of cancer cells in the systemic circulation of patients with solid tumors was recognized more than a century ago [8], it is only in the past two decades that the ability to isolate CTCs has enabled their molecular characterization and their use as prognostic and response biomarkers.

CTCs provide the opportunity to assess the biological features of cancer repeatedly during the evolution of the disease, enabling clinicians to react quickly to treat the patient with the most suitable specific targeted therapy.

Here, we review the clinical studies to date on the use of CTCs as biomarkers of cancer development and discuss the promise of CTC analysis to guide clinical decision-making and to aid targeted drug development.

## Identification of circulating tumor cells: technical aspects

**Figure 1:**
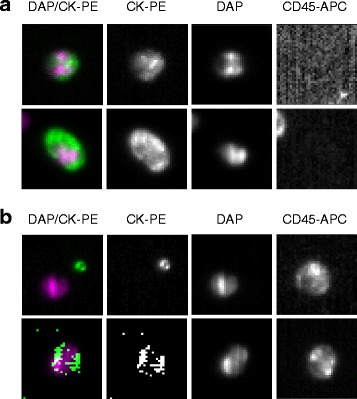
**Visualization of circulating tumor cells (CTCs) after isolation with the CellSearch semiautomated system (Janssen Diagnostics). (a)** Two CTCs from a blood sample from a cancer patient. Both cells have an oval morphology and have a diameter >4 μm. The nucleus is visible with DAPI (DAP) and they are positively stained for epithelial markers (cytokeratins, CK) but not for CD45 (a leukocyte marker). **(b)** Two ‘events’ identified as CTCs by the automated system but that were discarded by the trained human operator as each does not fulfill the characteristics of a CTC. APC, allophycocyanin; PE, phycoerythrin.

Methods to capture CTCs from blood rest on their differential physical or immunologic characteristics. The basis for affinity-binding systems used for CTC ‘enrichment’ is the selection of cells expressing certain antigens, such as epithelial cell-adhesion molecules (EpCAMs), and the discard of those cells expressing antigens that are known to be absent on epithelial cells but expressed by other blood cells, such as leukocyte-expressed CD45. Alternatively, CTCs can be isolated based on their distinct physical (size or deformability) or electromagnetic properties [9-11]. The enriched CTC population is then evaluated using an imaging system and, although counting can be completely automated, this step usually requires a certain degree of input from a human operator (Figure 1).

In order to implement a biomarker into clinical practice, it is crucial to obtain analytical validation (assay sensitivity, specificity and predictive value must be robust and constant) and evidence of the clinical significance of any results obtained. In 2011, the semi-automated CellSearch system (Janssen Diagnostics) became the first US Food and Drug Administration-cleared assay for quantitative analysis of CTCs, based on its high reproducibility [5] and the prognostic value demonstrated in prostate, breast and colorectal cancer studies [12–14]. This platform selects CTCs from blood samples according to size, presence of a visible nucleus and markers of epithelial origin (EpCAM, CD8, CD18 and CD19 positivity and absence of CD45) [15].

There are several promising capture platforms in development; a separate article in this special issue comprehensively reviews the advances in isolation and characterization of CTCs [16]. Potential advantages offered by some of the methods in progress include: first, a lack of a need for protein-based enrichment with the Epic Sciences platform, which retains all nucleated cells and permits their study with a high-definition scanner, allowing for a more complex characterization of CTCs and direct observation of ‘clusters’ of cells [17]; second, capturing higher amounts of CTCs with microfluidic platforms (CTC-chips) [18,19]; and, third, the possibility of continuous capturing of CTCs from blood by using detectors inserted into the patient´s veins or apheresis-based approaches with subsequent *ex vivo* CTC isolation [20,21]. This last method enables the screening of CTCs over larger volumes of blood, which could be relevant for monitoring residual disease in early stages of cancer, when low counts are expected.

## Clinical utility of circulating tumor cell counts

### Prognostic value

The number of CTCs present in the bloodstream of patients with metastatic cancer is prognostic for overall survival in several tumor types, with robust evidence reported for prostate, breast and colorectal cancer [12-14].

**Figure 2:**
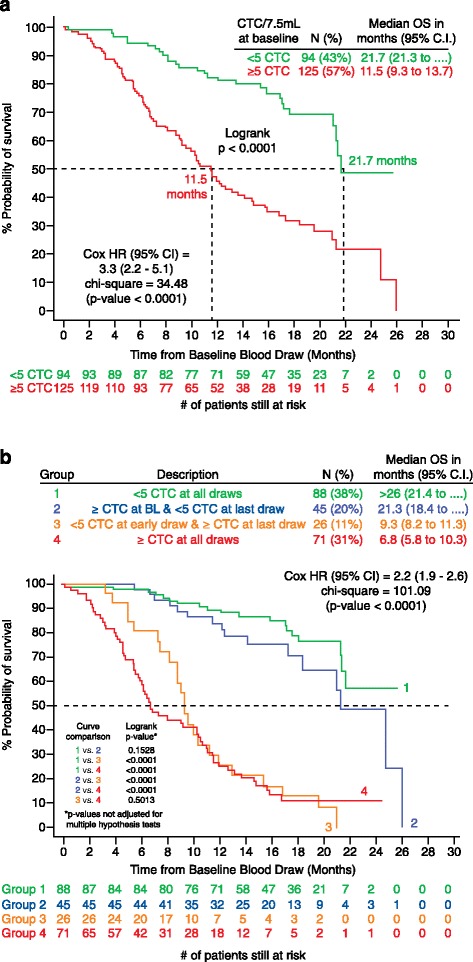
**Prognostic and predictive value of circulating tumor cell (CTC) counts in metastatic castration-resistant prostate cancer (mCRPC). (a)** Survival curves (Kaplan-Meier) of a group of 276 patients with mCRPC who were about to start a new line of chemotherapy, showing how patients with <5 CTCs per 7.5 ml blood (‘favorable’ CTC count; green line) had a better prognosis than the group of patients with higher counts (red line). **(b)** Differential survival curves representing outcomes for patients according to the change in their CTC counts after receiving treatment, which show that patients with a conversion from an ‘unfavorable’ to a ‘favorable’ count during the response to treatment (blue line) experience longer survival than those without a CTC drop (red and orange lines). The green line is the survival curves for those patients who have low CTC counts at the beginning to that study and whose counts remained low throughout the study. OS, overall survival; HR, Haza-ratio; CI, confidence interval. Reprinted from [13] de Bono JS *et al. Clin Cancer Res* 2008, 14:6302-6309. (Copyright by American Association for Cancer Research).

In metastatic castration-resistant prostate cancer (mCRPC), the prognostic value of baseline CTC counts was first assessed in the IMMC38 trial, a study including 164 patients about to start first-line chemotherapy (81% received docetaxel, and the remainder received a docetaxel-containing regimen). A high count of CTCs at baseline (defined as ≥5 CTCs in 7.5 ml blood) was associated with a significantly shorter survival compared with having low counts at baseline (11.5 versus 21.7 months; *P* < 0.0001). CTC counts were better indicators of survival than levels of prostate-specific antigen (PSA) (Figure 2) [13,22]. Further clinical trials in mCRPC have confirmed these findings [23].

A study by Cristofanilli established the prognostic value of CTC counts in advanced breast cancer. Patients with metastatic breast cancer were tested for CTCs at the time of starting a new line of treatment (either hormonal, chemotherapy or other treatments; 83 patients were assessed before the first line of systemic treatment, whereas 92 had received previous therapies). Patients with ≥5 CTCs per 7.5 ml blood had the worst progression-free survival (2.7 months versus 10 months; *P* < 0.001) and overall survival (10.1 months versus >18 months; *P* < 0.001). In a multivariate analysis including several molecular and clinical prognostic factors, CTC counts were a strong independent prognostic factor [24].

In the setting of metastatic colorectal cancer, the prognostic significance of CTCs was prospectively evaluated in 430 patients before starting a new line of chemotherapy. Patients were stratified based on CTC levels of ≥3 versus <3 per 7.5 ml blood. Patients in the ‘unfavorable prognosis’ group had shorter overall survival (9.4 months versus 18.5 months; *P* < 0.001) and progression-free survival (4.5 months versus 7.9 months; *P* = 0.0002) [14]. Pilati and colleagues also concluded that CTCs were prognostic in patients with liver-confined metastasis from colorectal cancer candidates to receive savage liver surgery, suggesting that CTCs might help to stratify the management of this population [25].

Clinical validation of CTC counts as prognostic biomarkers is being pursued in advanced stages of other tumor types, with promising results reported in lung [26,27], melanoma [28], head and neck [29] or pancreatic cancer [30].

Moreover, CTCs have also been demonstrated to inform prognosis in earlier stages of the disease. A large study analyzed their presence in 735 patients with colorectal cancer undergoing surgery with curative intent. CTC enrichment selected cells expressing carcinoembryonic antigen, cytokeratin 19, cytokeratin 20 and/or CD-133. Time to relapse and overall survival was significantly poorer for those patients with detectable CTCs [31].

In early stages of breast cancer, CTCs were detected in 21.5% of 2,026 patients after surgery and before the start of adjuvant chemotherapy. The presence of CTCs in this population was an independent prognostic factor for disease-free and overall survival [32].

Overall, CTC enumeration is a strong prognostic tool in patients with advanced cancer and can help in patient stratification. In earlier stages of cancer, there is high hope that CTC enumeration could monitor residual disease after radical treatment, although the counts in this population would be generally low, and the analysis would require a platform with very high sensitivity.

### Markers of response

Changes in CTC counts as a result of anticancer treatment have also been shown to be a reliable marker of response to treatment in different clinical settings. In mCRPC, the predictive value of changes in CTC counts and the correlation with survival have an enhanced relevance owing to the challenge of evaluating the response to treatment by standard radiological parameters as most of the metastatic disease is confined to the bones [33,34]. The IMMC38 study, mentioned above, was the first to correlate changes in CTC counts with clinical outcome in mCRPC: those patients with a baseline high CTC count that converted to a low count after chemotherapy had significantly better outcomes than those patients whose counts remained high despite the treatment (Figure 2) [13]. These findings were later validated in the randomized phase III study that led to the approval of the CYP-17 inhibitor abiraterone (Zytiga, Janssen Biotech) [35].

Similarly, conversion from an unfavorable to favorable CTC count after 4 weeks of treatment (using a threshold of 5 CTC per 7.5 ml blood) is a predictive biomarker of response to first-line therapy for metastatic breast cancer (progression-free survival of 9.4 months compared with 4.9 months for patients with high CTC counts at baseline that remained high on therapy). Moreover, drops in CTC counts upon treatment correlated with improved overall survival [12]. CTCs offer an early readout of response to treatment (4 weeks) instead of having to wait until the appearance of changes in radiological biomarkers, which might happen later; a study in 138 women with metastatic breast cancer receiving different treatments compared the performance of CTCs (after 4 weeks of treatment) and radiological assessments (after 10 weeks), concluding that CTCs were a more robust marker of patient outcome, and with a lower rate of inter-reader variability in interpretation of results (0.7% versus 15%) [36].

The value of CTC counts on treatment as an independent marker of response is also well established in metastatic colorectal cancer [14]. The study from Cohen and colleagues confirmed that patients with low CTC counts upon therapy had extended progression-free and overall survival, and this predictive value was maintained when assessed at different time-points throughout the therapy. The cut-off value in this case for the favorable versus unfavorable prognosis groups was 3 CTCs per 7.5 ml blood.

Almost every study judging CTC counts as response markers in different cancer types has simplified the enumeration to a dichotomist variable (high or low count) by using particular thresholds in different tumor types instead of considering CTC counts as a continuous variable; a combined analysis of CTC counts from 111 patients with metastatic breast cancer and 185 with mCRPC who participated in the IMMC-01 and IMMC-38 clinical trials [13,24] supports this strategy [37]. A study in mCRPC patients suggested that a relative decrease (30%) from baseline CTC counts remains predictive of response in univariate and multivariate analyses [38].

## Molecular characterization of tumors from circulating tumor cells

### Molecular profiling of circulating tumor cells

CTCs represent a valuable resource for studying the molecular underpinnings of cancer in individual patients. CTC analysis can support the delivery of precision anticancer treatments, as specific biomarkers of response to targeted drugs can be appraised.

Genomic- and proteomic-based assays can be used to analyze CTCs and detect the presence or absence of key signaling oncogenic aberrations. For instance, cytogenetic studies based on fluorescence *in situ* hybridization have been used to describe the variability between CTCs, tumor metastasis and primary prostatic cancers in copy-number aberrations for the androgen receptor and presence of TMPRSS2-ERG fusions and loss of the tumor suppressor gene *PTEN* (encoding phosphatase and tensin homolog) [39].

An example of how transcriptome studies in CTC analysis can be clinically applicable was the assessment, by RNA *in situ* hybridization studies, of how relative changes during treatment in the expression of epithelial and mesenchymal markers in CTCs from 11 patients with metastatic breast cancer correlated with response and prognosis [40].

As a result of recent progress in next-generation sequencing techniques, it is now also possible to perform whole-genome and transcriptome amplification from single cells, such as CTCs [41]. Lohr and colleagues were able to isolate single CTCs expressing PSA (assessed by low-coverage single-cell RNA sequencing) from patients with metastatic prostate cancer and sequenced the whole-exome [42] despite the limited input material that can be obtained from single CTCs, which remains the main challenge for such studies [43]. In the field of transcriptome assays, Ramsköld and collaborators developed a platform for efficient and robust single-cell RNA sequencing. In a cohort of samples from patients with advanced melanoma (a tumor of non-epithelial origin and therefore in which EpCAM-based cell selection is not useful), these investigators were able to analyze the transcriptome from single CTCs expressing melanoma markers [44].

### Treatment selection and monitoring of drug resistance

As cancer subclonal composition and driver mutation landscapes can change through the outgrowth of drug-resistant subclones after exposure to systemic therapy, it might also be important to assess whether predictive biomarkers are present at the specific time of starting a new line of targeted treatment, contrary to testing biopsies taken at earlier stages of the natural history of the disease; a study on 254 patients with breast cancer was able to detect overexpression of human epidermal growth factor receptor 2 (HER2) in CTCs in almost one-third of patients who had no HER2 overexpression in the primary tumor [45]. This finding has tremendous clinical relevance as overexpression of HER2 is a clinically validated predictive biomarker of response to HER2-targeting therapies in breast cancer [46,47].

A proof-of-principle study in lung cancer detected activating mutations in the gene encoding the epidermal growth factor receptor (EGFR) in CTCs from 11 out of the 12 patients tested; these patients were receiving EGFR-targeting tyrosine kinase inhibitors, and interestingly some of these mutations in CTCs had emerged *de novo* (that is, were not present in the matched primary tumor), suggestive of temporal evolution and a putative acquired mechanism of resistance to therapy [48]. Analogous results in patients with metastatic melanoma receiving serine/threonine-protein kinase B-Raf (BRAF) inhibitors, colorectal cancers patients treated with anti-EGFR antibodies and patients with gastrointestinal stromal tumors during tyrosine-protein kinase Kit/alpha-type platelet-derived growth factor receptor (KIT/PDGFR)-targeting treatment [49-52] support performing real-time analysis of predictive biomarkers of response in cancer medicine, and minimally invasive CTC-based studies represent an advance compared with tumor biopsy samples for repetitive, real-time analysis.

Ideally, patients would start a targeted treatment, and molecular indicators of response or resistance would be assessed in CTCs and/or other circulating biomarkers repeatedly over the course of treatment, guiding clinicians regarding when to stop or switch the anticancer treatment. A recent and successful example of this strategy is a study that serially characterized CTCs from 58 patients with mCRPC while receiving either enzalutamide or abiraterone to study mechanisms of resistance; quantitative reverse-transcription polymerase chain reaction was utilized to interrogate the presence of splice variants of the androgen receptor genes in CTCs. Antonarakis and colleagues demonstrated that the splice variant 7 of the androgen receptor (AR-V7) is a predictive factor of enzalutamide or abiraterone failure and, more interestingly, that the appearance of AR-V7 during the course of treatment in patients with no AR-V7 expression at baseline might be a mechanism of acquired resistance to these drugs [53].

## Circulating tumor cell analysis to understand intratumor heterogeneity

Genetic intra-tumor heterogeneity (ITH) has been discovered across a wide range of solid tumor types. For example, exome sequencing of multiple tumor regions from clear-cell kidney tumors, the commonest type of renal cancer, revealed that, on average, only one-third of the somatic mutations and DNA copy-number aberrations detected were present in every region that was analyzed from an individual tumor [54]. Mutations or hypermethylation of the *VHL* gene and loss of heterozygosity of chromosome 3p were the only aberrations detected in all analyzed regions from each tumor, suggesting that these were early founder aberrations, whereas other molecular aberrations, including mutations in members of the phosphoinositide 3-kinase/mammalian target of rapamycin (PI3K/mTOR) pathway (that is, mTOR, TSC2, PTEN and PIK3CA) amenable to be targeted with specific drugs, were heterogeneous within individual tumors. Genetic ITH has also been identified across many other solid tumor types such as primary breast, gastric, bladder, prostate and pancreatic cancers, where various degrees of genetic ITH, ranging from intermixed subclones to spatially separated subclones, have been described [54-58].

Integrating ITH in individualized treatment selections remains an unmet clinical need in cancer medicine. Sequencing studies on CTCs allow heterogeneous mutations to be captured, enabling a more detailed picture of ITH and subclonal evolution in a single patient with a minimally invasive approach. It is envisioned that the analysis of subclonal heterogeneity could help clinicians to understand why cancer patients might not respond homogeneously in different metastases to treatment with a targeted drug and might eventually guide treatment selection.

Using CTCs for ITH studies facilitates the inference of subclonal structures. Genomic-profiling studies of individual CTCs isolated from patients with metastatic colorectal cancer using the CellSearch platform showed how the presence of some genomic aberrations in CTCs was indicative of their subclonal origin from specific areas of the original tumor, through array-comparative genomic hybridization and multiplexed targeted sequencing of 68 genes relevant to colorectal cancer [41]. CTCs also further permit the study of gene-expression signatures, including dynamic drug-induced changes [59]. The functional interrogation of CTCs might provide crucial insights into the phenotypes of heterogeneous tumor subclones.

## Circulating tumor cell studies to guide drug development

Pharmacodynamics (PD) studies are crucial in modern drug development to provide evidence for proof-of-mechanism in early-phase clinical trials and support ‘go/no go’ decisions about further development of new compounds. Minimally invasive access to CTCs from patients offers a unique opportunity to monitor the PD effect of drugs in phase I clinical trials [60].

Assessment of PD biomarkers in CTCs can be performed repeatedly over the course of treatment, which represents an advantage compared with the study of tumor biopsies. To serve as an example, Wang and colleagues developed a quantitative assay to monitor changes in nuclear levels of the DNA damage marker histone variant γH2AX in CTCs that they then tested in samples from 15 patients with different cancer types participating in diverse phase I trials [61]. Among them, an increase in the nuclear γH2AX of CTCs as a response to therapy was observed in five patients receiving DNA-repair targeting drugs (topotecan, cyclophosphamide and/or inhibitors of the poly (ADP-ribose) polymerase PARP). Later, a phase I study of the PARP inhibitor Niraparib (MK-4827; Merck/Tesaro) implemented quantitative (changes in CTC counts over the course of treatment) and qualitative assessment of CTCs (nuclear γH2AX expression) as exploratory endpoints to support preliminary signs of antitumor activity [62]. In this study of Niraparib, 21 patients with mCRPC were treated, with no radiological responses observed; however, several patients had significant drops in CTC counts, which were especially remarkable in the three patients with a time-to-progression of over 6 months.

**Figure 3:**
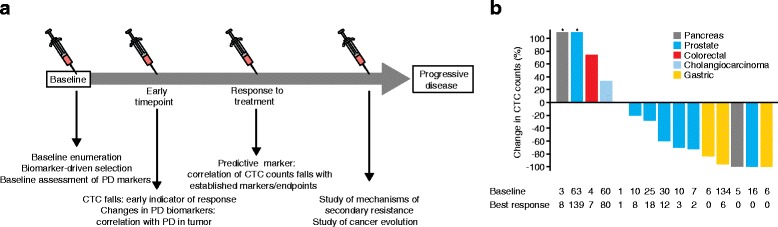
**Integrating quantitative and qualitative studies on circulating tumor cells (CTC) in early clinical trials. (a)** Schematic representation of how studying CTCs at different time-points during the treatment can provide information on drug effects (PD studies), discover mechanisms of resistance and also assess changes in CTC counts as predictive markers of responses. **(b)** Changes in CTC counts compared with baseline enumeration in patients participating in the phase I trial of the c-MET inhibitor ARQ-197. PD, pharmacodynamics. (Reprinted with permission from [63] Yap TA *et al. J Clin Oncol* 2011, 29:1271-1279. (Copyright by American Society of Clinical Oncology. All rights reserved).

Variations in CTC counts after drug exposure can also provide an easy and rapid readout of PD effects: the phase I trials of ARQ197 (a selective inhibitor of the hepatocyte growth factor receptor c-MET) in patients with different tumor types and EZN4176 (a second-generation antisense oligonucleotide to exon 4 of the androgen receptor) in prostate cancer assessed the changes in CTC counts in patients in parallel to the determination of the optimal doses of the drugs [63,64] (Figure 3). Precisely in the setting of phase I clinical trials, where patients with advanced stages of different tumor types and commonly a short survival expectancy are exposed to novel therapies, baseline CTC enumeration is of value as an independent prognostic factor and improves the performance of prognostic indexes used in clinical practice to select patients for these studies [65].

CTC analysis in early clinical trials can also be used to assess putative predictive biomarkers of response to novel targeted treatment and to provide proof-of-mechanism of novel compounds and a definition of biologically active doses. Hotspot mutations in PIK3CA were identified in CTCs from patients with either colorectal or breast cancer in two different studies [66,67]. Moreover, Kallergi and collaborators [68] were able to assess the activation of the PI3K-AKT-mTOR pathway in two groups of patients with early (*n* = 16) and metastatic (*n* = 16) breast cancer, reporting high expression levels of phospho-PI3K and phospho-AKT in CTCs from both populations. These results open the door to implementing such assays for patient selection in trials testing drugs against this important oncogenic signaling pathway, as has been done previously with tumor-tissue-based assays [69].

## Conclusions and future directions

CTC analysis is a validated method to study tumor characteristics and aid clinical decision-making. This minimally invasive approach facilitates repetitive analysis over time. Analysis can be purely quantitative, which has been proven to deliver prognostic and predictive information at different stages of cancer, or can involve the assessment of molecular biomarkers in CTCs. It is crucial that the novel isolation platforms currently in development are analytically and clinically validated to ensure biomarker integrity.

One caveat for CTC studies is whether the whole burden of disease contributes equally to the CTC pool or whether some subclones of the disease might be under-represented or absent in CTCs. Additionally, it is also important to remember that the CellSearch system and other enrichment platforms based on epithelial markers might fail to recognize cells shed from non-epithelial cancers such as melanomas or sarcomas, in which disease-specific technologic approaches and composite selection criteria might be needed [70], or cells that have lost expression of epithelial markers owing to undergoing an epithelial-mesenchymal transition, a central part of the invasion-metastasis process [71,72].

Molecular characterization of CTCs to guide rational treatment selection has direct applicability in clinical practice; it can help to overcome the inability of single biopsies to portray accurately the genomic landscapes of cancers and the limitations of multi-metastasis biopsy approaches. The possibility of regularly re-assessing evolving cancers is extremely useful for on-going treatment stratification and understanding primary and secondary drug-resistance.

Nevertheless, the exact role of CTC counts in routine clinical practice remains yet to be defined. On the one hand, the costs and complexity associated with the enrichment and enumeration process (including kits and operator time) are still high. On the other, a first clinical trial evaluating the benefit from an early switch on systemic treatment guided by changes in CTC counts after the first cycle of treatment in metastatic breast cancer recently failed to demonstrate significant benefit for this strategy compared with maintaining treatment until progression based on traditional endpoints [73]. However, the strong prognostic and predictive value demonstrated for CTCs in different tumor types and stages of the disease warrants further evaluation as a surrogate marker of survival endpoints in other clinical scenarios, which could change not only clinical practice but also the way clinical trials are designed. Thus, overall, quantitative and qualitative studies of CTCs represent a promising tool to advance toward the delivery of precision medicine to cancer patients.

## Abbreviations

APC: Aallophycocyanin
AR-V7: Splice variant 7 of the androgen receptor
BRAF: Serine/threonine-protein kinase B-Raf
CI: Confidence interval
CTC: Circulating tumor cell
EGFR: Epidermal growth factor receptor
EpCAM: Epithelial cell-adhesion molecule
HER2: Human epidermal growth factor receptor 2
HR: Haza-ratio
ITH: Intra-tumor heterogeneity
mCRPC: metastatic castration-resistant prostate cancer
OS: Overall survival
PD: Pharmacodynamics
PDGFR: Platelet-derived growth factor receptor
PE: Phycoerythrin
PSA: Prostate-specific antigen
